# Desalination technologies, membrane distillation, and electrospinning, an overview

**DOI:** 10.1016/j.heliyon.2023.e12810

**Published:** 2023-01-09

**Authors:** Monis Bin Abid, Roswanira Abdul Wahab, Mohamed Abdel Salam, Lassaad Gzara, Iqbal Ahmed Moujdin

**Affiliations:** a Center of Excellence in Desalination Technology, King Abdulaziz University, PO Box 80200, Jeddah, 21589, Saudi Arabia; b Department of Chemistry, Faculty of Science, Universiti Teknologi Malaysia, 81310, UTM Johor Bahru, Johor, Malaysia; c Department of General Studies, University of Prince Mugrin Al Munawara, Saudi Arabia; d Department of Chemistry, Faculty of Science, King Abdulaziz University, P.O Box 80200, Jeddah, 21589, Saudi Arabia; e Enzyme Technology and Green Synthesis Group, Universiti Teknologi Malaysia, 81310, UTM Johor Bahru, Johor, Malaysia; f Advanced Membrane Technology Research Centre (AMTEC), Universiti Teknologi Malaysia, 81310, UTM Johor Bahru, Malaysia; g Department of Mechanical Engineering, King Abdulaziz University, P.O. Box 80200, Jeddah, Saudi Arabia

**Keywords:** Desalination, Membrane distillation, Membrane, Biofouling, Electrospinning

## Abstract

Water is a critical component for humans to survive, especially in arid lands or areas where fresh water is scarce. Hence, desalination is an excellent way to effectuate the increasing water demand. Membrane distillation (MD) technology entails a membrane-based non-isothermal prominent process used in various applications, for instance, water treatment and desalination. It is operable at low temperature and pressure, from which the heat demand for the process can be sustainably sourced from renewable solar energy and waste heat. In MD, the water vapors are gone through the membrane's pores and condense at permeate side, rejecting dissolved salts and non-volatile substances. However, the efficacy of water and biofouling are the main challenges for MD due to the lack of appropriate and versatile membrane. Numerous researchers have explored different membrane composites to overcome the above-said issue, and attempt to develop efficient, elegant, and biofouling-resistant novel membranes for MD. This review article addresses the 21st-century water crises, desalination technologies, principles of MD, the different properties of membrane composites alongside compositions and modules of membranes. The desired membrane characteristics, MD configurations, role of electrospinning in MD, characteristics and modifications of membranes used for MD are also highlighted in this review.

## 1 Introduction

Populations all around the world are agitated by the significant issue of water scarcity. By 2050, it's anticipated that about 6 billion people would experience this scarcity. Only 30% of the freshwater that makes up the hydrosphere is suitable for human consumption. Deep subterranean water, glaciers, and ice caps make up the remaining 70% of the planet [1]. Saline water makes up a sizable portion of the hydrosphere on Earth, so desalinating saline water to remove its salts and minerals offers a promising method for extracting fresh water from saline water [2].

There are two fundamental technological approaches to desalinating water: thermal and membrane approaches as shown in Fig. 2 [3]. Reverse osmosis (RO), nanofiltration (NF), multiple-effect distillation (MED), mechanical vapor compression (MVC), and membrane distillation (MD) are a few of the technologies that have been applied to the desalination process over time [4]. In the process of thermal desalination, water from seawater is evaporated using heat energy, leaving behind only pure water after condensation. Because of the Middle East's plentiful fossil fuels, poor quality seawater, and appropriateness for electricity cogeneration, thermal desalination systems are commonly utilized there [5]. Even while thermal approaches generate more water with the total dissolved solids ranging from 5 to 50 parts per million, the system has the drawback of requiring a significant quantity of energy to complete the removal process, leading to high operational costs [6].

Thermal-based procedures like MED, multi-stage flash distillation (MSF), and MVC need a lot more energy than membrane methods like forward osmosis (FO), RO, and electrodialysis (ED). While salt content affects the energy requirements for membrane processes, salt concentration has no impact on the energy needs for thermal desalination systems [7]. The substantially lower energy requirements of membrane technologies versus thermal ones are probably their most well-known benefit. While MSF uses between 10 and 16 (kWh/m^3^) and MED uses between 5.5 and 9 (kWh/m^3^), RO energy requirements remain at 3 to 4 (kWh/m^3^) for seawater or decrease to 0.5 to 2.5 (kWh/m^3^) for brackish water [6].

When pressure is applied to the saltwater feed solution during the desalination process that is larger than just its osmotic pressure or the minimal pressure that prohibits the intake of pure water by osmosis, water is ejected through a semipermeable membrane [8]. RO is not advised for desalinating highly concentrated salt solutions because of the increasing osmotic pressure which also causes an accelerated flow of salt across the membrane [9]. An alternative desalination method called membrane distillation, which combines heat and membrane processes, is more suited for working with extremely salty solutions, especially for solutions having salinity between 70 and 300 g of salt per kilogram of solution [10].

The Bodell's membrane distillation (MD), which was developed in 1963, is one of the more modern methods for desalinating seawater that has been studied by scientists [11]. Due to its high salt removal rate, lack of considerable pretreatment, and low energy usage using waste heat as a source, it is a new method for desalinating water [12]. It is a non-isothermal membrane-based method for desalinating seawater that makes use of a suitable hydrophobic microporous membrane. By utilizing the temperature difference (thermal gradient) created across the membrane, MD is driven by a vapor pressure difference. High rejection of dissolved and non-volatile species results from water molecules evaporating and diffusing through the membrane before condensing on the permeate side (almost 100%). Low temperatures and pressures are suitable for MD to operate [13]. MD's requirement for heat might also be satisfied by solar energy that is renewable and waste heat [14]. Moreover, MD has been progressively used to treat wastewater with high nitrogen-ammonia levels. For instance, when DCMD was used to treat animal and poultry breeding effluent, over 90% of the ammonia-nitrogen was rejected [15,16]. It even reached 99% after anaerobic digestion effluent was pretreated with acidification [17].

**Fig. 1:**
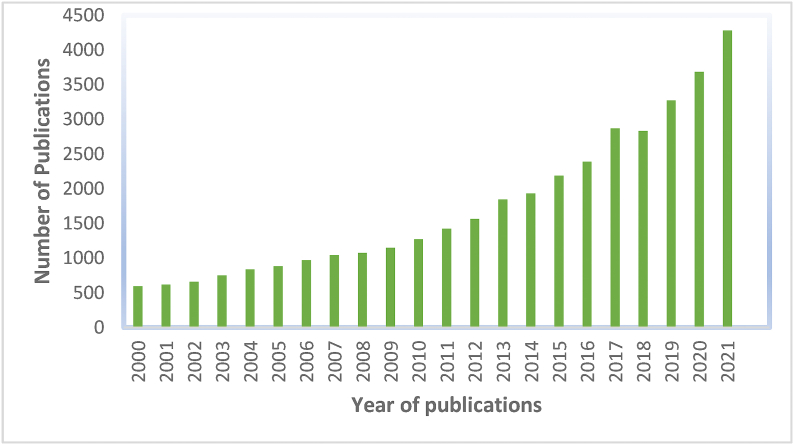
The number of publications on Membrane distillation (searched with the key word “membrane distillation”) from Science Direct Journal).

There are several advantages to MD over conventional distillation methods, including lower running temperatures, higher non-volatile element rejection capabilities, lower hydraulic pressure than the RO process, and a smaller overall footprint [18]. The number of publications for MD has significantly increased since the start of the twenty-first century, as shown in Fig. 1. Despite increased research efforts, MD is still not widely used in industry [19].

**Fig. 2:**
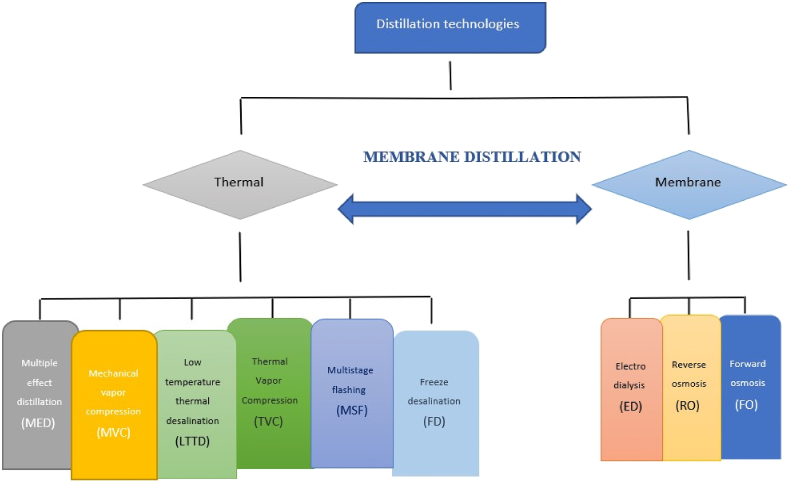
Classification of desalination technologies.

The aim of this review article is to critically analyze the limitations of technologies used in MD. Moreover, the role of membranes and their limitations will also be discussed in depth to reach the point of getting the answer to the question, “Why is the MD not being used in industrial scale?"

## 2 Desalination technologies

Technically, there are two primary methods for desalinating water: the thermal method and the membrane method [3]. MED, also known as multiple effect evaporation (MEE), and MSF desalination, are examples of thermal techniques. The former, known as MED, is the most traditional desalination method today and is also one of the most thermodynamically effective. It is made up of many cells that operate at lowering pressures and temperatures while recapturing heat from steam condensation. However, because it is regarded as being extremely dependable and sturdy, MSF is the thermal technique that is most frequently employed globally. Water is heated in a brine heater after passing through a number of heat exchangers in this procedure. It goes through several stages of abrupt decompression that result in vapor (“flashes” into steam) [20].

Reverse osmosis is the most popular technology when it comes to membrane technologies, or those that use semi permeable membrane to extract solutes from water (RO). The use of high hydraulic pressure in RO is to overcome the osmotic pressure of the water [21]. The semi-permeable membranes' small hole sizes allow the solvent (H_2_O) to flow freely but obstruct the passage of solutes that are trapped on the membrane's pressured side. Purified water and a very salty solution are obtained from the RO process (brine). The recovery rate (percentage) is the ratio between the end desalinated water and the original water intake, and it is often higher than 60% for commercial systems [22].

Forward osmosis (FO) separates various concentrations of solutions for water penetration by creating an osmotic pressure difference over a semipermeable membrane. When two different concentrations of solutions are kept on the two sides of a semipermeable membrane as a result of the osmotic pressure differential, water can pass through the membrane from the feed solution to the concentrated solution (draw solution). The draw solution is directed toward the membrane support layer, and the feed solution is directed toward the membrane active layer. Similar to pressure-retarded osmosis, the draw solutions are situated in the reverse direction [23]. FO is one example [2,24] that opeartes at a minimal pressure than RO, hence needing less energy. Membrane distillation (MD) [2,14] in which highly salinized water can be treated using a thermal separation process with microporous membranes, but this technology wasn't widely used until it was introduced in the 1960s [24]; however as a commercial approach, it is currently manifesting itself as a promising technology. Aquaporins, carbon nanotubes, nanoengineered membranes, and ion concentration polarization are only a few examples of the innovative membrane and material types that are the focus of desalination research and development [2,21].

Thomas Bartholinus (1680), who first proposed that freshwater could be obtained by melting ice made from seawater, is credited with inventing freeze desalination (FD), a technique for treating water that involves separating freshwater from saline solution in the form of ice crystals, followed by melting. The year 1950 marked the first commercial application of freeze desalination [25]. The direct freezing method using butane as the refrigerant for saline water desalination was first proposed by Karnofsky, Steinhoff, and Weigandt in 1950 [26].

Thermal vapor compression is similar to the Mechanical vapor compression system. A thermal steam compressor replaces the mechanical compressor [27]. By exploiting the temperature gradient between two bodies of water, the low-temperature thermal desalination (LTTD) method allows hotter water to escape at low pressures and condenses the resulting freshwater using colder water to produce high-quality freshwater [28]. In addition to being able to treat other types of feedwaters outside just seawater (such as brackish water, groundwater, or even wastewater), membrane technology also has the advantage of being modular, which enables future scale expansions or decreases in accordance with demand trends. Better energy efficiency and minimal economic expenses are further goals of experimental methods. Data collected from Ref. [29] Table 1 [29], [30], [31], [32], [33], [34], [32], [35], [36], [37], provides details of capacities, consumption of energy (kWh m^−3^), and cost (USD m^−3^) of various desalination technologies like MSF,MED,MVC,TVC,MD, ED,SWRO, BWRO, and FO. This table shows that MD is a quite economical process with capacity of 30,000,000 l/d, consumption of energy3–22 (kWh m^−3^) and cost 0.66(USD m^−3^).

**Table 1:** Lists various desalination techniques, their energy requirements, and the cost per unit of generating water [29].

Desalination techniques	Capacity	Consumption of energy (kWh m^−3^)	Cost (USD m^−3^)	References
MSF	73,646,658 l/d	21–59	4	[30]
MED	600,000 l/d	15–57	1	[31]
MVC	-	7–15	-	[32]
TVC	-	1.5–2.5	0.87–0.95	[33]
MD	30,000,000 l/d	3–22	0.66	[34]
ED	81,600 l/d	1–3.5	-	[32]
SWRO	80,000 l/d	3–6	0.2–0.7	[35]
BWRO	-	0.5–3	0.53–0.99	[36]
FO	-	10–68	0.6	[37]

## 3 Principles of membrane distillation

**Fig. 3:**
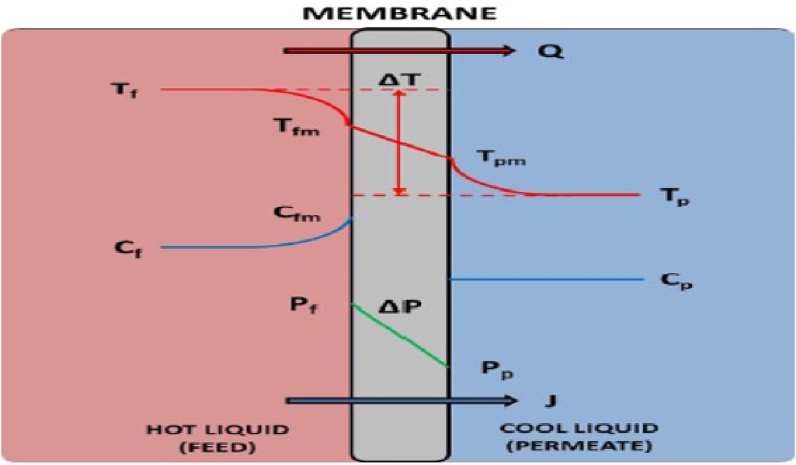
(Adapted from earlier research [40] with permission from the Royal Society of Chemistry; copyright 2019). T_f_ stands for “temperature of feed solution,” T_fm_ for “temperature on membrane surface in feed solution stream,” Tp for “temperature in the permeate stream,” Tpm for “temperature on membrane surface in permeate stream,” C_f_ for “concentration in feed solution stream,” C_fm_ for “concentration on membrane surface in feed solution side,” Cp for “concentration in permeate stream,” P_f_ for “hydraulic pressure of feed solution,” and Pp for “permeate stream (hydraulic pressure of permeate).

It is possible to process mine water, wastewater, radioactive wastewater, saline water, saltwater, and reverse osmosis brine using the membrane distillation (MD) method [38,39]. The most interesting technology for desalinating seawater is MD. Common advantages of MD include lower hydraulic pressure than the RO process, excellent non-volatile element rejection capacity, lower operating temperature, and a smaller environmental impact than conventional distillation processes [18]. The hydrophobic membrane is positioned between feed and permeate during the MD process. Only vapor molecules are able to pass across the membrane as a result of its hydrophobic property **(**Fig. 3 [40],).

Therefore, using MD process, it is possible to turn wastewater and saline water into better quality water and a concentrate that has the components as the mother liquid but is concentrated. The primary force behind this severance is a vapor pressure gradient (P = P_f_ - Pp) caused by the difference in temperature (T = T_fm_ - T_pm_) between hot feed (f) and cold permeate (p) [41]. Fig. 3 shows the concentration, hydraulic pressure, and temperature conditions for direct contact membrane distillation (DCMD).

The concentration polarization (C_P_) and temperature polarization (T_P_) lead to distinctive real concentrations and temperatures at the membrane surface between bulk solution and permeate [42,43], ending in a lack of driving force and mass transfer. These tolerate heat and mass transfer in the membrane's vicinity at the boundary layer. As a result, detailed awareness of these occurences is necessary for the effective management of permeate side in MD/MDC. Direct contact membrane distillation, vacuum membrane distillation, air gap membrane distillation, and sweep gas membrane distillation are examples of membrane distillation processes. Every setup has drawbacks and benefits, and are described below as configurations of MD [40].

## 4 Configurations commonly used in membrane distillation

**Fig. 4:**
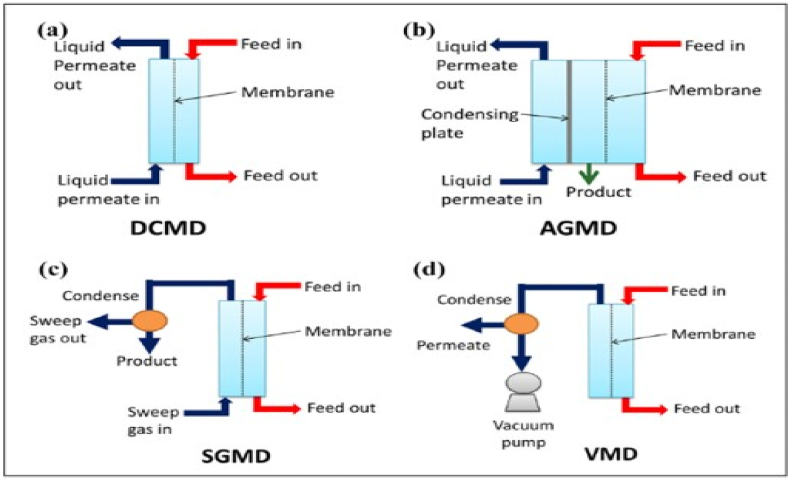
(a) DCMD process, (b) AGMD process, (c) SGMD process (d) VMD process [47] with permission from Royal Society of chemistry, copyright 2019.

The maximum rejection rates at maximum permeate flux have been attained when membrane distillation has been tested in various configurations for the desalination and generation of better quality water from saline water [44]. Various configurations were researched for getting back the heavy metal-contaminated subsurface waters [45] and for the cleanup of textile wastewater and pharmaceutical waste [46]. Direct contact membrane distillation (DCMD), air gap membrane distillation (AGMD), sweeping gas membrane distillation (SGMD), and vacuum embrane distillation (VMD) are the four configurations that make up MD processes as shown in Fig. 4 [47], [47].

### 4.1 Direct contact membrane distillation (DCMD)

In DCMD, the surface of the hot membrane side is in direct contact with the hot solution (feed). Then, the feed side is connected to the permeate side, water vapor is transported and condensed. Due to the difference in vapor pressure, the vapor gradient moves the water vapor across the membrane (Fig. 4). Unless otherwise specified, DCMD refers to the default MD setup [48]. This arrangement, which incorporates several NPs types (such as SiO_2_NPs) onto MD membranes, has been thoroughly studied for its use in the purification of various types of fluids (such as oilfield and salty), juice concentration, and the extraction of metals and ammonia [49].

Table 2 [13, [50], [51], [52], [53], [54], [55], [56], [57]], suggests applications and operating conditions of different omniphobic membranes. Omniphobic silica sand ceramic hollow fiber membrane was used for NaCl (35 g/L) and humic acid (10 mg/L). The feed and permeate temperature were maintained at 80 °C and 10 °C. 100% salt rejection was obtained with permeate vapor flux 49.41 kg/m2∙h. Omniphobic mullite hollow fiber membrane (HFM) was applied for humic acid (10 mg/L) and 35 g/L NaCl solution. The feed and permeate temperature were set at 65 °C and 20 °C. 100% salt rejection and permeate vapor flux 4.32 kg/m^2^∙h were achieved by using this omniphobic membrane. Omniphobic membrane (FZnO-PVDF) used for 3.5% NaCl solution with SDS concentration (<0.05 mM). The feed and permeate temperature were set at 60 °C and 20 °C to get 99.9% of salt rejection, and permeate vapor flux 12 kg/m^2^∙h. Omniphobic membrane (glass fiber + ZnO + florination), employed for 1 M sodium chloride with SDS concentraions 0.1–0.3 mM). The feed and permeate temperature were set at 60 °C and 20 °C. 100% of salt rejection with permeate vapor flux 11.4 kg/m^2^∙h was attained. Omniphobic PVDF nanofibrous membrane was exploited for 3.5 wt% NaCl solution with 10 mL mineral oil and 1 mL Tween 80. The feed and permeate temperature were set at 60 °C and 20 °C. This omniphobic membrane was used to get 100% of salt rejection, and permeate vapor flux of 11.4 kg/m^2^∙h. Omniphobic membrane of PVDF and Silica nanoparticles was used for SDS:hexadecane:NaCl at the concentration ratio of 240:2400:10000 mg/L in water. The feed and permeate temperature were set at 60 °C and 20 °C. Stable permeate flux and decreased conductivity on the permeate side were achieved. Omniphobic PVDF membrane with silica nanoparticles, use for cooking wastewater. The feed and permeate temperature were set at 60 °C and 20 °C to get permeate vapor flux 18.30 kg/m2∙h. Omniphobic PVDF membrane with silica nanoparticles, used for CaSO4 solution and synthetic Casein solution. The feed and permeate temperature were set at 70 °C and 20 °C. Used for stable flux and scaling and fouling resistance behavior.

**Table 2:** Omniphobic membranes with different composition used in DCMD.

Membrane	Application	Operating Conditions	Results	Reference
Omniphobic silica sand ceramic hollow fiber membrane	NaCl (35 g/L) and humic acid (10 mg/L)	The feed and permeate temperature were maintained at 80 °C and 10 °C	100% of salt rejection, permeate vapor flux 49.41 kg/m^2^∙h	[50]
Omniphobic mullite hollow fiber membrane (HFM)	humic acid (10 mg/L) and 35 g/L NaCl solution	The feed and permeate temperature were set at 65 °C and 20 °C	100% of salt rejection, permeate vapor flux 4.32 kg/m^2^∙h	[13]
Omniphobic membrane (FZnO-PVDF)	3.5 wt% Sodium chloride solution with SDS concentration (<0.05 mM)	The feed and permeate temperature were set at 60 °C and 20 °C	99.9% of salt rejection, permeate vapor flux 12 kg/m^2^∙h	[51]
Omniphobic membrane (glass fiber + ZnO + florination)	1 M sodium chloride with SDS concentraions 0.1–0.3 mM)	The feed and permeate temperature were set at 60 °C and 20 °C	100% of salt rejection, permeate vapor flux 11.4 kg/m^2^∙h	[52]
Omniphobic-hydrophilic janus membrane	3.5 wt% NaCl solution with SDS concentration (0.1–0.4 mM)	The feed and permeate temperature were set at 65 °C and 25 °C	99.9% of salt rejection, permeate vapor flux 16.62 kg/m^2^∙h	[53]
Omniphobic PVDF nanofibrous membrane	3.5 wt% NaCl solution with 10 mL mineral oil and 1 mL Tween 80	The feed and permeate temperature were set at 60 °C and 20 °C	99.4% of salt rejection, stable permeate vapor flux	[54]
Omniphobic membrane of PVDF and Silica nanoparticles	SDS:hexadecane:NaCl	The feed and permeate temperature were set at 60 °C and 20 °C	Stable permeate flux and decreased conductivity on the permeate side	[55]
	At the concentration ratio of 240:2400:10000 mg/L in water			
Omniphobic PVDF membrane with silica nanoparticles	Cooking wastewater	The feed and permeate temperature were set at 60 °C and 20 °C	permeate vapor flux 18.30 kg/m^2^∙h	[56]
Omniphobic PVDF membrane with silica nanoparticles	CaSO_4_ solution and synthetic Casein solution	The feed and permeate temperature were set at 70 °C and 20 °C	Stable flux and scaling and fouling resistance behavior	[57]

Lee et al. have successfully attained the thermal efficiency of 0.73–0.87 by a countercurrent cascade which is a significant improvement in membrane distillation [58].

### 4.2 Air gap membrane distillation (AGMD)

The hot side of the membrane surface is in direct contact with the feed solution in this configuration. Membrane thickness and air gap length are added to determine the overall length of vapor diffusion. Between the hot membrane surface and the condensation side, stagnant air is introduced (Fig. 4). The air gap allows the water vapor to enter the membrane's condensation chamber [59]. This configuration has been used in several investigations, including one that used a PVDF nanofibre membrane with an almost 150° contact angle to filter harmful metals out of water [45,60].

### 4.3 Sweeping gas membrane distillation (SGMD)

During the SGMD procedure, a non-reactive gas is employed to sweep the vapor out of the compartment of the membrane's permeate and into its compartment of condensation (Fig. 4). A moveable gas barrier also helps with mass transfer and reduces heat loss. The fundamentals of the process, the features of the membrane, the materials used for the membrane, the membrane modules, the process variables, flux improvement, transport method, and polarization process have all been outlined by Onsekizoglu [61].

### 4.4 Vacuum membrane distillation (VMD)

On the permeate side of the membrane, a vacuum is produced in a VMD configuration. Driving the water vapor outside the barrier causes it to condense (Fig. 4). The loss of heat is greatly reduced in this setup [62]. In order to evaluate membrane wetting resistance and flow increase, Ka et al. investigated employing a mechanically strong and superhydrophobic SiO_2_-altered PVDF nanofiber membrane in VMD. In solar-powered devices, VMD has also been utilized to recover water from contaminated solutions [63,64].

Data from Ref. [65] Table 3 highlights advantages and disadvantages of various membrane configurations. DCMD used for sea water desalination and arsenic removal from aqueous solutions has high permeate flux but has the disadvantage of conductive heat loss. AGMD is a simple process and has low heat loss but its flux is lower than DCMD and VMD. VMD is considered to be used at commercial scale because of high fux but it is a complex process and poses a high pore wetting risk. SGMD used for brackish water desalination and waste water treatment is used for reduction of barrier for mass flow but it has disadvantage of maximum risk of water polarization.

**Table 3:** advantages and disadvantages of different configurations [65].

MD configuration	Advantages	Disadvantages	Application Area
Direct contact membrane distillation (DCMD)	Permeate flux high can be up-scaled	Conductive heat loss	Seawater desalination
			Arsenic removal from aqueous solution crystallization
			Treatment of dye effluents
Air gap membrane distillation (AGMD)	Low heat loss	Flux is smaller than DCMD and VMD	Concentration of fruit juices; separation of azeotropic mixtures; seawater desalination; and VOC elimination
	Simple process		
	Temperature polarization risk is low		
Vacuum membrane distillation (VMD)	Permeate flux high	High pore wetting risk	Desalination of sea water. Treatment for an alcoholic beverage. Chemicals used in aroma recovery. Wastewater treatment for textiles
	Can be considerd at comercial scale	A complex process	
Sweeping gas membrane distillation (SGMD)	Reduction of the barrier for the mass flow	Temperature polarization risk maximum	Brackish water desalination and azeotropic mixture separation. Water treatment for waste. Removing VOC

## 5 Membrane modules

### 5.1 Flat sheet

**Fig. 5:**
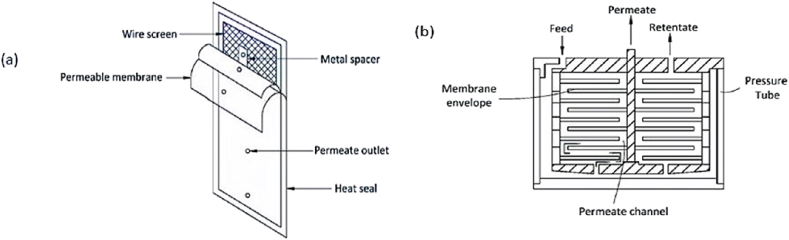
(a) Model of plate and frame membrane module and (b) Plate and frame membrane module operation [68] with the permission of Emma, copyright from IWA publishing 2018.

Over the past 50 years, this configuration has consistently been the most thoroughly explored membrane module configuration in membrane distillation [66]. Usually, plate and frame modules were constructed using flat sheets or plates. The blank spaces between the two rectangular frames were filled with these flat sheets. The four MD configurations (DCMD), (AGMD), (VMD), and (SGMD) are compatible with this membrane module [67]. These modules maintain spacers in between the pairs of flat sheets. Because of their simplicity, they are typically utilized in laboratories. These modules are related to desalination and water purification [14]. For water treatment and desalination, plate and frame modules are appropriate to MD [68]. Fig. 5 [68–72], show the modules and their operation.

**Fig. 6:**
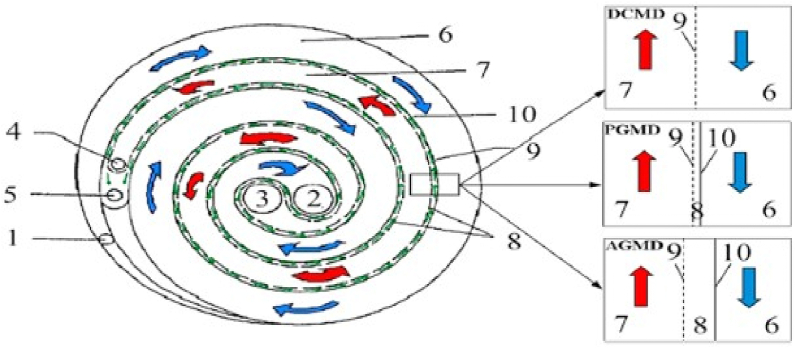
(With permission from ref. [69] is a schematic illustration of a spiral-wound module with one pair of main flow channels, (Elsevier 2017).

**Fig. 7:**
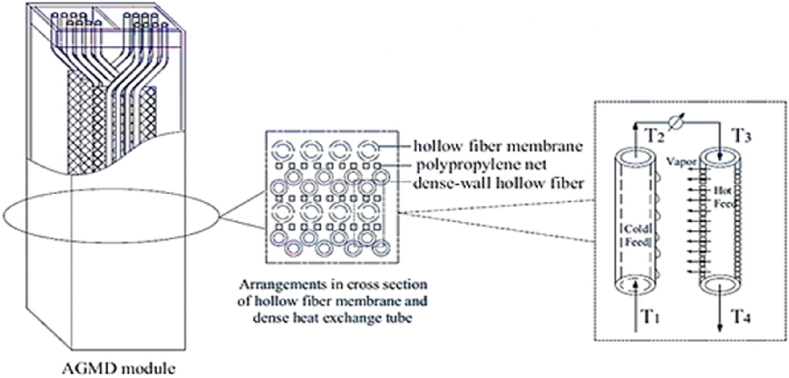
Scheme of the HF module with internal heat recovery [70] (with permission from Elsevier 2014).

**Fig. 8:**
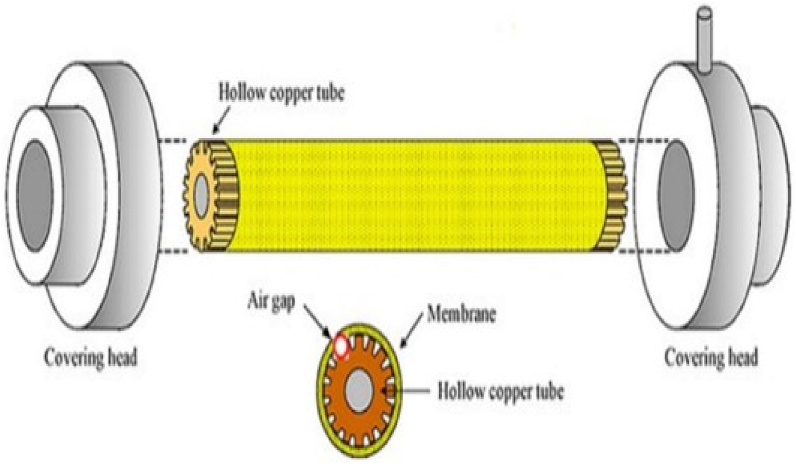
Tubular module in AGMD with permission from ref. [71].

**Fig. 9:**
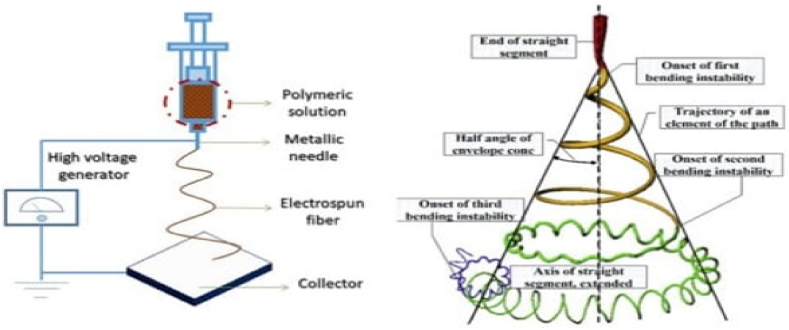
Schematic representation of the ES process with permission from ref. [72].

These modules were frequently employed in the research industry for saltwater desalination, employing MD technology, due to its benefits. Particularly, these modules are simple to construct, connect, use, test, and clean. The removal or replacement of damaged membranes is likewise easy and transparent. This module is widely used throughout laboratories to examine how operational factors and membrane properties affect the permeate flux or energy efficiency of membrane distillation [73]. Additionally, Out of all membrane module types used under identical working conditions, the MD technology that used a flat sheet membrane provided the maximum flow [74].

### 5.2 Spiral wound

In MD, brackish and seawater were desalinated using spiral-wound membranes [70]. The flat sheets and spacers are designed to be packed and coiled around the pierced collection area with the help of this module as shown in Fig. 6. The spiral membrane was made up of a cover that was coiled and twisted around a perforated permeate collection tube, a mesh spacer, a permeate carrier, and a support layer for the membrane. The membrane is traversed axially by the feed solution, and the filtrate exits through the collection area [14].

It uses a reasonable amount of energy, packs well, and is not prone to fouling. The two types of microfiltration procedures used for MD are crossflow and dead-end flow. Crossflow involves driving the feed tangentially to the membrane with treated water that then flows through the membrane, with the feed solution being cycled [68,75]. Spiral-wound membrane modules first emerge only two decades after membrane modules initially started playing a significant part in the MD. After the membrane distillation process gained prominence, only a very small percentage, roughly 2%, of these modules were used in MD research. Additionally, there wasn't much enthusiasm early on for using these modules in MD applications. A spiral membrane in particular was used in 1% of the investigations. The drawbacks of this membrane module study led to its limitations [76]. One of them caused the difficulty in cleaning or replacing when membranes are fouled: the spiral-wound membrane construction is made by rolling different membranes and support layers. Thus, the fouling problem could affect the spiral-wound membrane module. Additionally, permeate flow in the MD system using the AGMD module has inundated the air gap, necessitating a change in the MD design [66].

### 5.3 Hollow fiber

Studies on utilizing hollow fiber membranes in MD technology have grown more and more alluring in recent years [66]. Inside-out flow and outside-in flow are the two different types of flows in the hollow fiber membrane design used in the MD study. The internal and external flows did not mix because the membrane's material is hydrophobic; instead, a distinct boundary easily forms on the hollow surface of the fiber that makes up the membrane. The feed water went through the membrane's fiber (membrane element) in the direction of the outside-in flow as water vapor. The mass transfer was driven by the difference in pressure at the contact surface. Salts and other contaminants from the feed water were drawn through the membrane, gathered outside, and then removed using the bottom exit pipe and concentrated stream. Outside vapor entered via the thin membrane of hollow fibers [67]. The hollow fibers are fixed and arranged in the tubular shell of this module. Feed passes through the shell, while filtrate permeates the membrane. The studies of Laganà et al. [77] and Fujii & Kigoshi [78] in contrast to Elimelech et al., who employed capillaries to treat saline water and polypropylene membrane, they used this module to treat alcohol and apple juice [79]. These modules have a high packing density and low heat usage, making them useful. These modules' drawbacks include their increased propensity for fouling, which necessitates maintenance and occasionally replacement after damage [75].

Wang and Chung noted that this type's two primary flaws were its poor permeate flux and weak mechanical qualities [80]. Because the membrane's fibers must be secured into its cover, one of the main limitations of MD systems is that membrane cleaning is practically unregulated [14].

### 5.4 Tubular membrane

Tubular membranes had also been investigated for the MD process to desalinate seawater, treat brackish water, and handle wastewater [81,82]. This membrane is mostly composed of ceramic, PP, PVDF, and PTFE, and was used in the DCMD, AGMD, and VMD MD designs. This module was made out of a hydrophobic membrane and a shell [14]. Within the parallel cylinders of both cold and hot fluid, the tube-shaped structure is maintained. This module has some crucial characteristics, including a low susceptibility to fouling, a good contact area, and ease of maintenance. Additionally, it is taken into account for commercial applications. Although it can be used for MD as well, it has certain drawbacks, including a poor packing density and high operating expenses. In several MD setups, including DCMD, VMD, and AGMD, ceramic membranes are highly helpful for treating aqueous NaCl because they reject salts at a rate greater than 99% [14].

Studies on tubular membranes received less attention at the beginning of MD technology than those on flat-sheet membrane modules or hollow fiber membranes. Only 15% of studies worked on tubular membranes at first, but by the time MD technology was fully developed, that number had fallen to just 5%. This drop might be caused by this module's relatively low packing density (approximately 300 m^2^/m^3^) [14]. The permeate flux of the hollow fiber membrane was lowered to 20.48%, while that of this membrane declined by 7.33% when the salt content rose from 0 to 3 g/L of NaCl. As the salt concentration increased from 3 to 50 g/L of NaCl, the yield of outlet water of the tubular membrane was only declined by 2.7%; but, with the hollow fiber membrane, it was decreased to 3.6% [82]. Table 4 [66,67,74,84,85], [74,84,86,87], [88–92], [91,93], summarises from benefits and drawbacks of different membrane modules. According to this flat sheet module is simple to produce, use and assemble. It is simple to remove or replace it. Maximum flow in the MD process is obtained by using flat sheet module. However it has low mechanical strength and its packing density is very low. Effective packing density of spiral wound membrane is 300–1000 m^2^/m3 and is energy saving module. Also it has low-temperature polarization but it is extremely vulnerable to fouling. The maximum packing density in the case of hollow fiber membrane is 3000 m^2^/m3 and is capable of working at very high pressures (above 100 bars). It has greater effective surface area per volume. It does not use much energy. Its disadvantages are low flux permeation, poor mechanical qualities and membrane fouling. Tubular membrane has high flow rates and has greater surface area to volume ratio. It offers ow propensity to clogging and is simple to clean but its packing density is low. Its operation is expensive and it offers minimal permeate flux.

**Table 4:** Lists the benefits and drawbacks of MD modules.

Membrane Module	Advantages	Disadvantages	Reference
Flat sheet	•Simple to produce, assemble, use, test, and clean. •From this setup, damaged membranes are simple to remove or replace. •The maximum flow was obtained utilizing a flat-sheet membrane in the MD procedure. •To enhance the membrane area, numerous flat-sheet membranes can be put in the same membrane frame. •Compatibility with all four MD setups •Under the same operating conditions, the MD process using a this membrane produced the maximum flow among the other membrane module types.	•Packing density is very low (effective membrane area per unit volume is between 100 and 400 m^2^/m^3^). •Low mechanical strength. •The membrane support layer, which is required and has a significant impact on the membrane distillation process.	[66,67,74,84,85]
Spiral wound	•Effective packing density of 300–1000 m^2^/m^3^. •Reasonable amounts of energy are used. •Low-temperature polarization	•When fouling occurred, replacing or cleaning the membranes was challenging. •Minimal permeability •Extremely vulnerable to fouling.	[74,84,86,87]
Hollow fiber	•The maximum packing density is 3000 m^2^/m^3^. •Capable of working at very high pressures (above 100 bars). •Greater effective surface area per volume. •Used in numerous different industries, including wastewater treatment, artificial kidneys, liquid-liquid extraction, and desalination. •Does not use much energy. •Less susceptible to temperature polarization due to high mass and thermal transmission efficiency.	•low flux permeation. •poor mechanical qualities. •Membrane fouling is difficult to control. •Implementing the replacement of broken fibers was extremely challenging, which contributed to the high cost.	[[88], [89], [90], [91], [92]]
Tubular	•Permission for high flow rates. •The tubular membrane's surface area is significantly greater than its volume ratio. • Low propensity to clogging and simple to clean	•300 m^2^/m^3^ is a low packing density. •The complete module must be changed in the case of membrane wetness since the shell and tubes were entangled. •High cost of operation. •Minimal permeate flux.	[91,93]

## 6 Characteristics of membrane distillation membranes

The properties of the membranes are of utmost importance in defining their abilities in MD systems since MD relies on the membrane process. Low heat conductivity, low fouling rate, high LEP, high permeability, pretty long efficiency, thermal stability, chemical stability, and superior mechanical strength are all desirable characteristics in a membrane for MD. There may be a trade-off between certain membrane properties that influence the above-mentioned aspects. It is essential to understand that these characteristics are painstakingly managed to ensure that each feature reaches its ideal value. The significance of these properties, the characteristics of the membranes that influence them, and the relationships between trade-offs are the main topics of the following subsections.

### 6.1 Liquid entry pressure

It is common practice to estimate the hydrophobic membrane's wetting resistance using LEP measurement, which refers to the minimal hydrostatic pressure that needs to be applied to a liquid in order to enter and wet the membrane [94]. At the site of penetration, the applied pressure produces a force that is larger than the hydrophobic membrane's repelling force. High LEP is therefore required to prevent the feed fluid from penetrating the membrane and perhaps wetting the pores completely or partially [95]. To provide high flux, excellent permeate quality (high salt rejection), and long-term membrane performance, pore wetting prevention is necessary. With low membrane surface energy and a high contact angle with water, high hydrophobicity is produced, resulting in high LEP. Through Eq. (1) Wenzel's theory explains how contact angle and surface roughness are related [96].

(1) $$\cos\;\theta =\frac{r\left(\gamma_{sv}-\gamma_{sl}\right)}{\gamma_{lv}}$$

where γsv, γsl, and γlv are the relative interfacial tensions between solids and liquids, solids and vapors, and liquids and membranes, respectively, and surface roughness factor r. Franken and his colleagues [97] suggest that LEP is influenced by pore size and other factors, among others. by Eq. (2)

(2) $$LEP_{w}=\frac{-2B\gamma_{L}\;\cos\;\theta }{r_{\max}}$$

where liquid surface tension γL; θ is the CA between the membrane and liquid; and rmax is the maximum pore radius of the membrane, B is the dimensionless geometrical factor that accounts for the inconsistencies of the pores (B = 1 for cylindrical pores).

### 6.2 Thermal conductivity

To maintain a significant heat gradient that acts as a significant driving force for the movement of water vapor across membranes, a low thermal conductivity is essential. A membrane with high thermal conductivity can lose a significant amount of heat through conduction, exacerbating temperature polarization and plunging the thermal gradient. As a result, less water vapor permeates the membrane when the vapor pressure gradient is lower. The thickness of the materials, and membrane porosity can all have an impact on its heat conductivity. Compared to ceramic membranes, membranes which are made of polymers show a lower heat conductivity (0.1–0.5 Wm1 K1) [7,98]. Thinner membranes show extensive conductive heat loss, which is why they have greater thermal polarization [99]. Due to the lower thermal conductivity of the air inside the holes, high porosity reduces thermal conductivity while increasing membrane permeability [1]. Note that permeability also reduces with thickness, in addition to heat conductivity.

### 6.3 Permeability

A lot of water vapor permeates the membrane in a short amount of time. As shown by Eq. (3), big pore size, high porosity, tiny pore tortuosity, and thin membranes can all theoretically work together to produce a high molar flux [100].

(3) $$N\propto \frac{\left\langle r^{\propto }\right\rangle \varepsilon }{\tau \delta }$$

In the equation, N = molar flux, r = average pore radius for diffusion, membrane porosity *ε*, membrane tortuosity τ, and membrane thickness δ. MD also prefers the distribution of a narrow size (uniform distribution). Big holes and high porosity create a considerable overall surface area to allow evaporation and the passage of water vapor, whereas thin membranes have a low mass transfer barrier to the transit of water vapor [101]. However, for practical applications in MD, larger pore sizes and thinner membranes do not necessarily translate into higher molar flux. Large pores and thin membranes can result in lower LEP and significant conductive heat loss, respectively, reducing flux. An ideal membrane, thus, has an optimum pore size and thickness and high porosity to maximize the effective mass transfer and strike a balance between high LEP and high permeability [12]. Porosity, pore size, and thickness should be between 80 and 90%, 0.5 and 0.6 μm, and 100 and 200 μm, respectively. A value of tortuosity that is close to zero is optimal [1].

### 6.4 Fouling rate

The process of fouling is the buildup of undesired materials on the surface of the membrane or in the pores of the membrane, which affects the membrane's functionality. While pores obstructed by these materials can result in mimimal pore diameters and decreased flux of permeate, the existence of particles on the surface of the membrane can boost the wettability of the membrane [102]. A low fouling rate guarantees uninterrupted water vapor movement and high flux. There are three types of foulants that are frequently found: calcium sulfate, calcium carbonate as inorganic foulants; oil as an organic foulant, and bacteria as biofoulant. Because of their hydrophilicity, inorganic foulants enhance the membrane's wettability, whereas organic foulants promote wetting by reducing the surface energy of the membrane [96]. Seawater can have organic compounds, making membranes employed in MD for desalination vulnerable to fouling, especially by hydrophobic chemicals. Fouling wets the membranes, which lowers the temperature of the feed solution on the membrane and lowers the thermal gradient. Additionally, fouling may lower pressure, which in turn increases thermal polarization [103]. Even though pressure-driven processes like RO have a lesser likelihood of fouling than MD, getting rid of the fouling in MD systems will allow for longer-term operation, which finally leads to a reduction in operational expense [104].

### 6.5 Thermal and chemical stability

The membrane should have superior thermal and chemical stability as these properties guarantee the membrane's long-term performance even in challenging conditions. Very reactive compounds that can be harmful to the surface of membranes with limited chemical stability can be found in feed solutions such as contaminated saltwater. Additionally, cleaning solvents and backwashing may expose the membranes to substances that could cause them to degrade [105]. The membranes' lifespan can be reduced by deterioration, but also results in permeate flux contamination, which is very undesireable in MD. The membrane's performance may suffer and its structure may be affected by operating temperatures that are too high and surpass the polymer's melting point. The heat stability of polymers like PTFE, PVDF, PES is higher than that of polyethylene (PE) or polypropylene (PP), making them better suited for MD applications [100].

### 6.6 Long-term performance

MD membranes should perform at a continuously high level during a lengthy lifespan. This is crucial to keeping MD competitive with alternative desalination methods and lowering the cost of renewing the membranes. The long-term effectiveness research of MD membranes has only been documented in a small number of studies, despite its significance. It has been shown that membranes with active layers or surface coatings may be more prone to damage during prolonged MD operation [106]. In long-term operations, flux and salt refusal often tend to decrease over time [1].

### 6.7 Mechanical strength

This quality is essential for the membranes to endure the heavy stress and pressure applied when operating an MD and assembling modules. Inadequate mechanical potency can cause membrane rupture and pore collapse, which compromises the membrane's long-term function. Because they cause the membrane to become mechanically weak, macrovoids are undesirable in membranes. There is evidence that sponge-like structures prevent the creation of macrovoids [107]. Greater mechanical strength an be achieved with thicker membranes, but permeability is reduced.

## 7 Electrospinning

William Gilbert first observed the development of a cone-shaped water droplet in the presence of an electric field in a study he did in 1600. This observation inspired him to develop the idea of electrospinning. Stephen Gray discovered the electrohydrodynamic atomization of a water droplet, which produced a very fine stream, around a century later. The first known electrospraying experiment was carried out in 1747 by Abbé Nollet, who showed that water could be sprayed as an aerosol while passing through an electrostatically charged vessel that was positioned adjacent to the ground. The behavior of charged droplets was then thoroughly investigated by Lord Rayleigh. In order to establish how many charges a liquid droplet could contain before ejecting liquid jets from the surface, he performed a theoretical calculation in 1882. High voltage is used in both the electrospinning and electrospraying processes to eject liquid jets, making them identical procedures. When electrospinning, the jet can be kept in a continuous shape to create fibers rather than breaking up into droplets (for the generation of particles) as with electrospraying [108].

### 7.1 Technique and process

J.F. Cooley and W.J. Morton gave patents for the electrospun (ES) procedure in 1900 and 1902, respectively. Formulas created an experimental tool for electrostatic force-based polymer fiber preparation in 1934. It was the first-time detailed volume synthesis of fibers in high-voltage static electricity, and it is now largely acknowledged as the start of the ES technique in fiber preparation. Simple manufacturing equipment, cheap spinning costs, a broad range of spinnable materials, and accurate and regulated processes are all benefits of ES technology [109,110]. These benefits have made ES technology one of the most widely used techniques for successfully preparing nanofiber materials. The electrospun nanofibers have several exceptional qualities, including flexibility, a tiny diameter, a big surface area, a high aspect ratio, and unique physiochemical properties [111].

The ES technique device is comprised of a high-voltage source device, an injection tube with microscopic needle, and a metal collector plate/roller. It is important to regularly regulate the humidity and temperature in the surrounding area. During the ES process, a high-voltage electrical force is supplied to the system of polymer-solvent (polymer solution or polymer melt). The polymer solution is then injected from the spinneret using a powerful high electric field to break through the surface tension of the solution. Electrostatic force and surface tension have the most effects on a polymer jet during the stretching process. The stretching of the fibers during this process is influenced by a variety of variables, including Coulomb repulsion force, electrostatic force, surface tension, viscoelasticity, gravity, and air resistance [112].

### 7.2 Principle of electrospinning

A liquid droplet is electrified to create a jet during the electrohydrodynamic process of electrospinning, which is followed by stretching and elongation to create fiber [108]. With a few exceptions, most electrospun membranes are created by running the solution through a spinneret during the electrospinning process. However, a high voltage is given so that the particles within the solution are charged, so providing a repulsive force, rather than using air or mechanical devices to create the extrusion force. At a critical voltage, the solution's surface tension is broken by the repulsive force, causing a jet to explode from the spinneret's tip. In contrast to conventional spinning, the jet is only stable near the tip of the spinneret before becoming unstable due to bending. The solvent evaporates as the charged jet moves faster toward areas of lower potential, yet the polymer chains entangle to keep the jet from disintegrating. This causes the growth of fibers. To capture the fiber, a grounded plate is typically utilized [114].

### 7.3 Engineering of electrospun nanofibers

The composition, structure, and properties of the nanofibers can be customized to target certain applications by adjusting the materials and electrospinning techniques. With the right post-processing, new nanomaterials with a fibrous morphology can be created from electrospun nanofibers. Usually, after stabilizing and carbonizing polymer nanofibers, carbon nanofibers can be easily manufactured. Selective removal of the polymeric component from composite nanofibers results in the retention of metal or ceramic nanofibers. To provide new capabilities, various nanoparticles can be put into the nanofibers. A nonwoven mat made of electrospun nanofibers is porous and has a sizable specific surface area by nature. The porosity and specific surface area of the resulting mat can be further increased with the addition of in-fiber pores. Additionally, the morphologies of the nanofibers can be beaded, core-sheathed, or hollow [115].

Electrospun nanofibers can be constructed into arranged arrays or hierarchical structures by adjusting the alignment and/or patterning. If necessary, the nonwoven mat can be joined together at the nanofibers' intersection locations to create interfiber connections. The electrospun nanofibers can be altered using a variety of physical and/or chemical techniques in order to change their porosity and pore diameters as well as add additional functional groups and components. Furthermore, a 3D structure can be created by expanding a thin nanofiber mat in the vertical direction [108].

### 7.4 Effects of various parameters on electrospinning

**Fig. 10:**
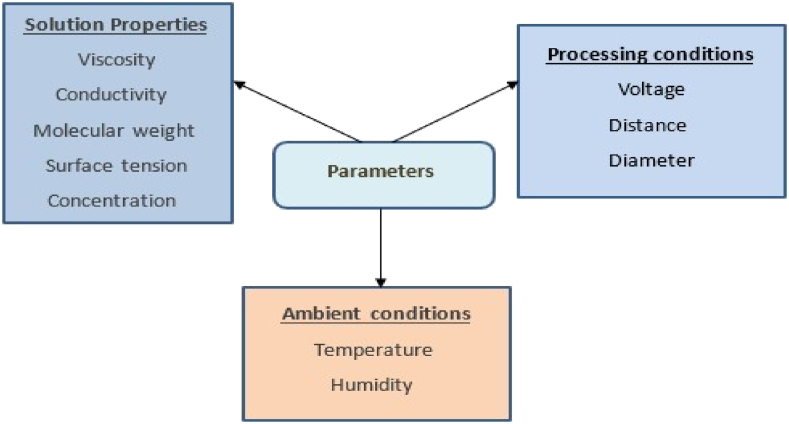
Parameters affecting electrospinning.

Various parameters affect the morphology of fibers during electrospinning and, as a result, the fibers may be broadly classified into solution parameters, ambient parameters, and process parameters as shown in Fig. 10. Solution parameters include molecular weight, concentration, viscosity, surface tension and dielectric effect of solvent applied electric field, tip to collector distance, collector types, needle diameter, and feeding or flow rate are some of the process characteristics. Each of these variables has a substantial impact on the morphology of the fibers produced by electrospinning, and with careful adjustment of these variables, one can create nanofibers with the appropriate morphologies and diameters [116].

Table 5 [117–127], elucidates effects of various parameters on fiber morphology in electrospinning. Among them viscosity is a dominant parameter that decides whether the solution will need to be electrospun or not. A too-low viscosity may interrupt polymeric filaments and droplets of polymer (electrospray), whereas a very high viscosity prevents the polymer from being extruded. Increase in concentration of polymer results in an increase in fiber diameter while increasing the molecular weight of the polymer results in reduction of beads. Increasing conductivity leads to decrease in fiber diameter. Greater surface tension results in destabilization of jets. Increase in voltage causes decrease in fiber diameter. The increasing voltage will accelerate the Electrospinning jet and this may result in a greater volume of solution drawn from the tip of the needle. Too large or too small distance causes generation of beads. High humidity results in circular pores on the fibers. Increase in temperature results in decrease in fiber diameter.

**Table 5:** The various parameters on electrospinning and their effects on fiber.

Parameters	Effect on fiber morphology	References
Viscosity	The dominant parameter decides whether the solution will need electrospun or not. A too-low viscosity may interrupt polymeric filaments and droplets of polymer (electrospray), whereas a viscosity that is too high prevents the polymer from being extruded.	[117]
Polymer concentration	Increase in fiber diameter with an increase in concentration	[118,119]
The molecular weight of the polymer	Reduction of beads with increasing the molecular weight of the polymer. When the molecular weight is too low, beads rather than fibers are more likely to develop.	[120]
Conductivity	Decrease in fiber diameter with an increase in conductivity	[121,122]
Surface tension	Greater surface tension results in destabilization of jets	[123]
Voltage	Reduction in fiber diameter as voltage rises. The increasing voltage will accelerate the Electrospinning jet and this may result in a greater volume of solution drawn from the tip of the needle	[124]
Distance	Generation of beads with too small and too large distance, the minimum distance required for uniform fibers	[125]
Humidity	High humidity results in circular pores on the fibers.	[125]
Temperature	Increase in temperature results in decrease in fiber diameter.	[126,127]

### 7.5 Electrospinning strategies and approaches for membrane fabrication

#### 7.5.1 Single-spinneret electrospinning

Due to its simplicity, this setup is the most popular. Since only one spinneret or nozzle is utilized—as implied by the name—only one syringe pump is often needed to push a single solution. Due to the ease of producing tiny to suitable sized nanofiber membrane samples, this is especially suitable for laboratory-scale experiments. Different membrane fiber architectures, morphologies, diameters, and functions can be generated depending on the management of parametric circumstances [128].

#### 7.5.2 Multi-spinneret electrospinning

It is feasible to produce hybrid nanofiber membranes using different component polymers instead of just single or mixed component nanofiber membranes using a single spinneret system during electrospinning. This can be accomplished by electrospinning two or more solutions concurrently onto a single collector surface using a dual or multi-spinneret electrospinning system [129]. Different configurations of multi-spinneret/nozzle electrospinning apparatus can be used in a lab setting. One involves using a collection of spinnerets that are circumferentially or side-by-side [130]. Due to the micro-sized smooth polystyrene (PS) fibers, this design resulted in a beaded nanofiber membrane that was extremely hydrophobic and had good mechanical integrity. The multi-spinneret setup is perfect for increasing electrospinning productivity [131].

#### 7.5.3 Coaxial electrospinning

Coaxial electrospinning is a variation on single jet electrospinning that uses a concentric tube or core-sheath spinneret to enable the electrospinning of two separate solutions simultaneously rather than a single spinneret with one solution [128]. Nanofiber that is coaxially electrospun has a core-sheath arrangement. Two solutions, either identical or dissimilar, are injected into each of the syringe containers using separate or a single syringe pump, and the coaxial spinneret is attached to the high voltage power source. Charges build up largely on the sheath layer in this system, where they behave as a single spinning jet and form a Taylor cone. Once the surface tension is broken, a stable jet is ejected. Because of viscous forces and contact friction, the sheath layer's shear stresses cause the core solution to drag. The core fluid assumes a conical shape and flies together with the sheath fluid in a core-sheath structure once it has been carried by the sheath fluid [129].

As long as the tip of the cone produces a stable jet with the core-sheath fully formed, bending and whipping instability with solvent evaporation follows until a solidified core-sheath fiber is created on the collector. The morphology and homogeneity of the resulting fiber are significantly influenced by the composition of the core and sheath layers [132]. However, the process becomes more difficult as a result of the additional solutions supplied because of the miscibility issue and the distinct solidification and conductivity behaviors of the two solutions. For solutions that are difficult to electrospin or for polymers that are difficult to dissolve, coaxial electrospinning is a promising option. These “challenging” polymers could be carried by either the core or the sheath layer. Hollow fiber membranes are also made with coaxial electrospinning [133].

#### 7.5.4 Melt electrospinning

Solvent-based solutions were utilized in the majority of electrospinning studies. Polymers that are difficult to dissolve, such as polytetrafluoroethylene (PTFE), polypropylene (PP), and polyethylene (PE), cannot be electrospun using traditional solution electrospinning because they cannot be dissolved in a solvent [134]. In many instances, different types of polymers are dissolved using organic solvents to create the desired viscosity and concentration for electrospinning. When employed in industry, some chemicals and solvents may be hazardous to human health as well as the environment and may also leave behind residuals. Melt electrospinning has also been taken into consideration in the goal of cleaner production [133].

Since more established spinning technologies are available for producing micron-size fibers, melt electrospinning is not a particularly viable strategy since it uses molten polymers, which have a higher viscosity (approximately 40–200 Pa-s compared to 5 Pa-s for most polymer solutions) [135]. As smaller fibers (about 1 μm diameter) can now potentially be formed in comparison to earlier studies having diameters up to 50 μm, the fabrication of molten electrospun membrane has recently become more and more popular. A non-mechanically drawn melt electrospinning process has been used to produce fibers as tiny as 270 nm [116]. Melt electrospinning has a number of benefits, including more uniform fiber creation and no requirement for solvent, which means no ventilation or exhaust is needed and no concern with leftover solvents. However, since the polymer must be melted, heat is frequently required, which raises the cost. Additionally, heat is produced throughout the process and must be removed. Melt electrospinning often has a Tip-to-collector distance (TCD) that is 3–5 cm shorter than solution electrospinning and a flow rate that is much lower [136].

#### 7.5.5 Designs of electrospun membrane

Nanofibers are one of the most intriguing and significant 1D nanostructures that can be used to create nonwoven membranes among the various nanomaterials. Electrostatic spinning, also known as electrospinning, has emerged as a novel and uncomplicated technology that can easily and simply generate nanofiber membranes, is cost-effective, and has the potential to be scaled up, providing genuine opportunities in industrial applications. There is no disputing that nanofibers are becoming increasingly popular for use as membranes, largely because of their distinctive properties. Electrospinning allows for the precise construction of nanofiber membranes by adjusting the process and material parameters. The high porosity and small pore diameters of the nanofibers allow them to specifically filter out contaminants in the air and water processes [133].

A recent advancement in the field of MD is the development of polymeric composite and nanocomposite membranes with superior properties to their pure equivalents. For MD, three polymeric membrane designs—flat, electrospun, and hollow fiber membranes have been reported. Electrospun membranes are shaped like nanofibrous mats [100]. Electrospun nanofibrous membranes can be utilized for MD or as a highly porous substrate for making RO and FO membranes in the desalination process. Due to their high porosity, adjustable pore size, and functionalized surface of the nanofibrous scaffold, these exceptional nanofibrous membranes outperformed traditional membranes. Thus, specialized desalination applications, such as the desalination of brackish water or seawater, can be conveniently targeted using electrospun nanofibrous membranes. Electrospun nanofibrous membrane has been used to obtain high penetration flux and high rejection ratio, demonstrating the technology's enormous potential. The increased mechanical characteristics and high rate of manufacturing of electrospun nanofibrous membranes specifically for desalination applications are two of the numerous obstacles that remain. We anticipate that all of these issues will be resolved in the future, when various desalination techniques will increasingly rely on nanofibrous membrane technology [137].

Electrospinning is a development of electrostatic spraying, which has been used for more than 270 years to produce aerosols from liquid drops. The rapid advancement of materials and technology that would enable the production of nanofibers on a large industrial scale suggests that electrospun membranes have a promising future. Nanofiber scaffolds and membranes will continue to have a wide range of applications in the medical, environment, water, energy, transportation, and food sectors. Its usage in water treatment as filters or a filter component is super creative. No one can dispute the fact that additional research is necessary to fully realize the potential of nanofiber membranes, but the direction of current research trends is the right one. Robustness, durability, a more uniform pore size distribution, and mechanical strength of nanofibers are some of these difficulties [128].

Recently, electrospun nanofibrous membranes have attracted a lot of interest. They have high porosity, a three-dimensional network of interconnected pores, repeatability, and other distinctive and promising characteristics that account for this [138]. The inaugural study on the application of an electrospun membrane for the MD process was reported by Feng and colleagues in 2008 [139]. The membrane sample in this investigation was made using PVDF. Desalination tests on salty water with a 6-wt percent NaCl solution were carried out using the AGMD method. The authors found a remarkable salt rejection rate of more than 98%. The mechanical resilience of electrospun nanofibrous membranes is one of their most challenging limits. Li and coworkers [140] have done investigation into the use of spacer fabrics and nonwoven fabrics as the backing layer to enhance the mechanical properties of nanofibrous membranes. Electrospun membrane samples were made using PVDF polymer. The authors investigated how the support layer impacted the characteristics of the membrane (e.g., permeability, porosity, morphology, pore size and pore size distribution, hydrophobicity, and mechanical durability). The highest water flux measured throughout the investigation was 49.3 kg/m^2^/h when the hot stream temperature was set to 80 °C. The novel composite membrane additionally demonstrated a respectable long-term desalination performance. In another work, Deka and coworkers [141] developed a novel electrospun membrane with superior wetting resistance for MD-based desalination. As an alternative to saltwater desalination, the authors of this study looked at the creation and use of perfluorinated membranes having superhydrophobic characteristics [80, 83].

Only a few research teams had used electrospun membranes for MD treatments prior to 2014. Tijing and his colleagues thoroughly reviewed these studies [142]. In a different review publication, Shirazi and colleagues also conducted an analysis of the literature that addressed the production and application of electrospun membranes from 2014 to 2017. Since then, there has been a growing trend in the development and application of nanofibrous membranes made using electrospinning for the treatment of water using MD methods [143].

#### 7.5.6 Electrospun membrane for water filtration and desalination

One of the most serious worldwide issues is the availability of clean water, and efforts are being made to find new sources and treatment methods to address this issue [144]. Even if conventional treatment technologies already exist, ongoing efforts are made to improve the efficiency and adaptability of the current separation techniques. Applications for filtration and desalination can benefit from nanofiber membranes' low basis weight, extremely porous structure (>80%), and controllable pore diameters [145].

**Fig. 11:**
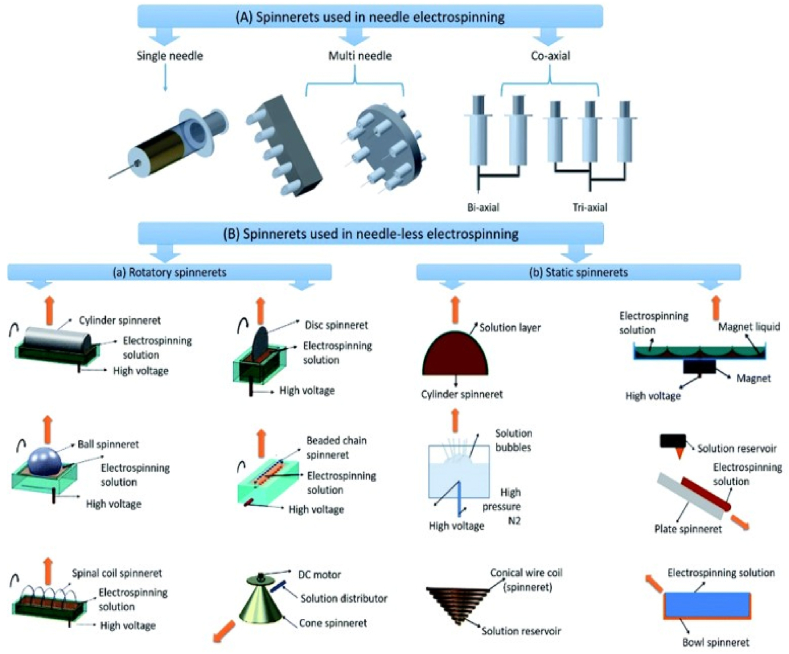
Spinnerets used in electrospinning (A) spinnerets in needle electrospinning. (B) Spinnerets in needle-less electrospinning; (a) rotatory spinnerets and (b) stationary spinnerets used in needle-less electrospinning. Reproduced from Ref. [148] with permission from American Chemical Society, copyright 2014.

Recent improvements in electrospinning have increased productivity while also greatly expanding the availability of nanofibers with a variety of morphologies. Nanofibers can now be oriented in the desired pattern. The co-axial and multi-needle spinnerets (Fig. 11A [148],) of the needle electrospinning setup, among other spinneret improvements, have made it possible to produce nanofibers more quickly and over a larger area. The advantage of using several needle spinnerets for producing ENMs with mixed nanofibers from various polymer solutions is that interference from the electrospinning process causes the nanofibers to mix [146]. By simultaneously electrospinning two or more different polymer solutions while feeding them through the spinneret, co-axial spinnerets have been utilized to create hollow, core-sheath, and composite nanofiber-based ENMs. By removing the fiber's core component, it is possible to acquire the ENMs of hollow nanofibers [147].

Needle-less electrospinning has become the best method during the past ten years for producing ENMs on a big scale and affordably. This method makes use of spinnerets in shapes other than needles, which are divided into rotatory spinnerets and static spinnerets. Fig. 11B illustrates the various rotatory and static spinneret kinds. The rotatory spinnerets include the rotating cylinder, rotating disc, rotating bead chain, rotating cone, rotating ball, and rotating spinal coil. High-quality nanofiber membranes are created as a result of electrospinning nanofibers upward while the other rotatory spinnerets, excluding the rotary cone, are partially submerged in the polymer solution. This prevents the polymer solution from dripping onto the nanofibers that have been deposited on the collector [149].

To support the ongoing manufacturing of nanofibers, spinnerets rotate continuously, supplying polymer solution to the electrospinning sites. The primary benefit of needle-free electrospinning is that several nanofiber jets are naturally initiated in the ideal locations on the spinnerets' surface where they are revolving [150]. These spinnerets remove clogging issues, allow for speedier production, and permit the deposition of nanofibers across a broader area. IME Technologies, Elmarco, and Inovenso Ltd. all have industrial needle-less electrospinning operations with a production capacity of several kilogrammes per hour. It primarily depends on the quantity of spinnerets used in electrospinning as well as the quantity of jet initiation sites that arise from the spinneret's free surface. However, with needle-free electrospinning, it is challenging to produce well-aligned, consistent, and varied shapes of nanofibers [151].

**Fig. 12:**
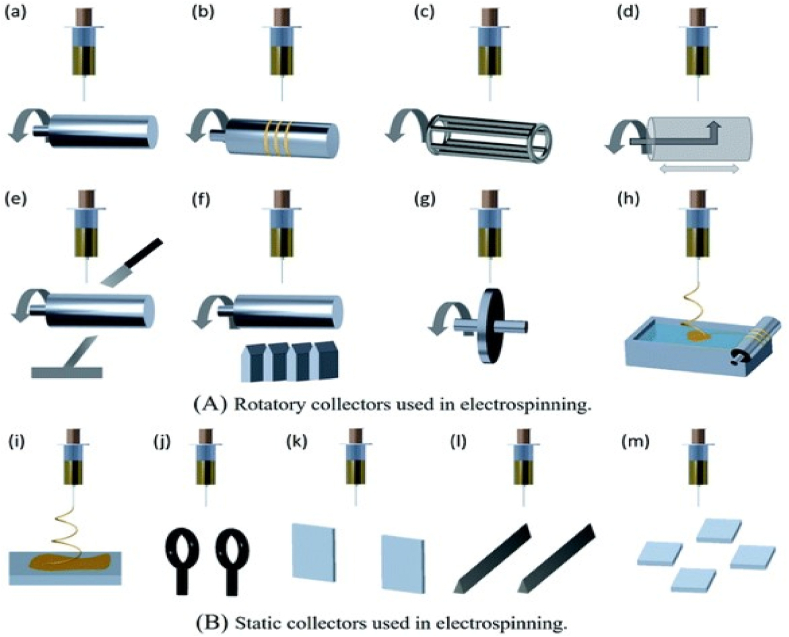
Different types of collectors used in electrospinning; (A) rotatory collectors (a–h), (B) static collectors (i–m); (a) rotating drum, (b) rotating drum with wrapped wire, (c) rotating wire drum, (d) rotating drum with sharp pin inside, (e) rotating drum with knife edge electrodes, (f) rotating drum with multiple knife edge electrodes, (g) rotating disk, (h) fiber collection using water bath, (i) plate collector, (j) parallel ring collector, (k) blade electrodes in line, (l) parallel electrodes, (m) array of counter electrodes [152] with permission from Royal Society of chemistry, copyright 2021.

Controlling the alignment of nanofibers in a desired direction is crucial for electrospinning technology. Different types of collectors have been created in order to better manage nanofiber alignment. According to Fig. 12 [152], the two main types of electrospinning collectors are rotatory collectors and static collectors. Large numbers of nanofibers can be aligned using a rotating drum collector. As a result of the high speed drum rotation, which can break nanofibers, it does not produce highly aligned nanofibers and calls for speed improvement [152]. The disadvantage of using a rotating wire drum collector is that, although it can produce highly aligned nanofibers, but at a certain thickness of deposition, the alignment and orientation of the fibres may not be suitable [153]. Large area aligned nanofiber mats can be created using a rotating drum with a sharp pin within; this sort of collector is typically employed to create thin mats of aligned nanofibers [154]. Depositing highly aligned nanofibers requires the employment of a rotating drum collector with knife edge electrodes. It is simple to create thick mats of deposited nanofibers due to the great alignment of the entire deposition [155].

To create highly aligned nanofiber membranes, a spinning drum collector wound with wire can be employed. In this collector, the region of nanofiber deposition can be adjusted by changing the wire's thickness [156]. Additionally, highly aligned nanofibers are obtained using a revolving disc collector. The disadvantage is that only a smaller area of aligned nanofiber mats can be produced, and the disc rotation speed must be regulated to prevent nanofiber breaking [157]. When employing a water bath collector, the polymer solution is electrospun into the water bath to solidify the nanofibers. Then, using a rotating drum collector, the twisted and aligned nanofibers are made into yarns [152]. A static collector, such as a plate collector, produces nanofiber mats that are randomly aligned, and a parallel ring collector makes it possible to create nanofiber yarns with twisted patterns. However, only a certain length of twisted yarn can be produced because one of the rings needs to be turned in order to produce twisted yarns [158]. Parallel electrode collectors generate well aligned nanofibers that are simple to transfer to various substrates. However, it is challenging to produce longer nanofibers and thicker deposition [159]. In certain circumstances, the nanofibers can be extended along the space between the collectors to create a unidirectional array of nanofibers by incorporating a small amount of magnetic nanoparticles into polymer solution and electrospinning under an external magnetic field [159].

#### 7.5.7 Electrospun membrane for membrane distillation

A hydrophobic membrane is used as a separating layer in the new thermally powered desalination method known as MD. Since there is currently no commercially available membrane for MD, numerous research organizations are constantly making significant attempts to synthesize or produce novel membranes [128]. Although commercial microfiltration membranes are suitable for MD, they still have poor permeation flux and are prone to wetting problems. Electrospun nanofiber membranes were taken into consideration for MD applications because of their high hydrophobicity, high porosity, and suitable pore diameters. Since the initial use of a nanofiber membrane for MD was reported in 2008, other research teams have produced nanocomposites and other distinctive hybrid designs, such as dual-layer and triple-layer, to construct and enhance the properties of nanofiber membranes [139], doing post-treatment methods such as hot-pressing and applying surface modification techniques. When compared to commercial membranes, findings in the literature generally showed improved permeation flux and salt rejection efficiency for nanofiber membranes; nevertheless, difficulties with long-term stability and fouling formation are still not thoroughly examined [160].

Self-supported tidy nanofiber membrane performs rather well but has a propensity to become wet over prolonged operation, especially if exposed to harsh feed water. As a result, numerous studies on the electrospun membrane for MD have implemented numerous modifications in an effort to enhance the characteristics of the clean nanofiber membrane [128]. Incorporating nanoparticles into or onto the nanofiber is one particular method for increasing the hydrophobicity of the membrane and even producing superhydrophobic surfaces. The nanoparticles are either surface modified or blended freely into the solution. Carbon nanotubes (CNTs), graphene, clay, TiO2, aerogels, silica NPs, boron nitride, and other NPs are employed. Even at higher feed salt concentrations (up to 70 g/L NaCl solution), the CNT/PVDF-co-HFP nanofiber membrane performed significantly better in direct contact membrane distillation (DCMD) in terms of flow and salt rejection than a commercial flat-sheet membrane [128]. Another strategy involves treating the nanofiber surface with plasma or UV light. In this method, the plasma treatment produces an omniphobic nanofiber surface by adding extra fluorinated surface. Due to larger hole diameters and smaller thickness, low LEP is a common problem of nanofiber membranes [161]. Low LEP indicates a significant propensity for pore wetting, which results in suboptimal MD performance. Many post-processing and design techniques have been used to address this problem, including hot pressing membranes, increasing nanofiber membrane thickness to reduce mean and maximum pore sizes, surface functionalizing membranes with hydrophobic molecules or nanoparticles to increase their hydrophobicity, etc [128].

## 8 Characteristics and modifications of membranes used for MD

Because MD depends on the membrane process, the characteristics of the membranes are of utmost importance in identifying their capabilities in MD systems. Some membrane characteristics that affect the factors mentioned above are occasionally traded off under certain circumstances, but these attributes are scrupulously controlled to guarantee that every one of the features attains its perfect value [162]. The following subsections focus on the characteristics and importance of the membrane's attributes, how each attribute affects Performance, and their trade-offs under certain circumstances. Among the popular membranes used viz. Polyvinylidene fluoride (PVDF), polyethylene (PE), polypropylene (PP), and polytetrafluoroethylene (PTFE) are hydrophobic polymers frequently employed in MD research. Another, albeit less prevalent, type of polymer utilized in MD is polydimethylsiloxane (PDMS) [1].

### 8.1 Requirements for membranes to be used in MD

High hydrophobicity, which prevents the liquid phase from penetrating the membrane's pores, is the primary quality of a membrane that is employed in MD. Practically, membranes consisting of polypropylene (PP), poly (vinylidene fluoride), and poly (tetrafluoroethylene) (PTFE) fully meet this need. The membranes must moreover have a liquid entry pressure (LEP) of at least 2.5 bar. LEP is the amount of pressure needed to allow fluid to pass through the membrane's pores. A further increase in pore diameter has a detrimental impact on the LEP value, hence membranes with pore diameters between 0.1 and 1 m are typically utilized in MD [163].. It has been stated that water flux and mass transfer are minimized with increasing the thickness of the membrane, while small thickness leads to heat losses that negatively affect the driving force of the process [164]. As a result, for a variety of applications, a membrane thickness of between 10 and 400 μm has been found ideal [165]. Another crucial element for membranes is porosity, with a high value increasing water flux. The porosity of membranes typically ranges from 30 to 90%, including membranes produced by the electrospinning technique [166]. Thus, the membrane to be used in MD should have the following properties.

1. A minimum LEP value of 2.5 bars.
2. Reduced pore wetting risk with narrow pore size distribution.
3. Membrane pore sizes between 0.1 and 1 μm are advised.
4. A membrane's ideal thickness should fall between 10 and 60 μm. Highly concentrated mixtures should be purified using thicker membranes (>60 μm).
5. The membrane's porosity ought to be as high as possible.
6. Membrane contact angles must be as large as feasible (>90).

### 8.2 MD membrane fabrication techniques

For MD, membranes can be manufactured using the procedures of stretching, phase inversion, and electrospinning [[167], [168], [169]]. A variety of membrane types have been created by combining various techniques. Zhu [170] fabricated a novel hydrophobic hollow fiber PTFE membrane by cold pressing, comprising extrusion, stretching, and sintering. Stretching is a method for creating membranes without the use of solvents. Micropores are created by extruding a polymer at a temperature just below its melting point.

This fabrication technology is less expensive than other ones. A film is created by extruding a polymer that has partial crystallinity stretched to the axis of crystallite orientation at a temperature below its melting point. This method allows for the production of membranes with high porosity (90%) and consistent porous structure [171]. A regulated transition of a polymer from a solution to a solid state is known as phase inversion, which is a phase separation process. The following steps make up fabrication via phase inversion: Prior to being cast on a plate, polymer pellets are first dissolved in a solvent to create a casting solution. Following that, the semi-liquid film is cast onto the plate and submerged in the bath to induce precipitation. Finally, a polymeric film with a symmetric or asymmetric structure is created [172].

It was suggested that electrospinning technology be used to create nanofiber membranes for MD [47,145,173]. An efficient technique for creating nanofibrous membranes with high porosity and roughness is electrospinning. Electrospun polymer membranes feature a large surface area to pore volume ratio [174]. Various polymers have been used to create electrospun membranes, including PVDF [175], PVDF–SiO_2_ [176], polystyrene (PS) [166], PTFE–polyvinyl acetate [177] and PVDF–HFP/SiNP [178]. It should be mentioned that electrospun membranes have been tested in a variety of configurations and are among the most widely used membranes. Kebria [178] and others suggested a technique for inserting dendritic patterns during electrospinning to increase the hydrophobicity of nanofiber PVDF membranes. By polycondensing the carboxyl groups of nitriloacetic acid and the hydroxyl groups of boehmite, dendritic structures were created.

SEM and contact angle measurements were used to evaluate the impact of various dendritic structure densities on membrane shape, elemental composition, and surface hydrophobicity. The contact angle rose from about 129 °C to 139 °C, and the salt rejection and water flux were 99% and 11 kg/m^2^·h and, respectively [179]. Feng and colleagues [180] were the first to implement the MD process for the purification of NaCl saline solutions using nanofiber PVDF membranes. The water flux and salt rejection were, respectively, 11,000–12,000 g/m^2^h and 99%. Prince et al. [181] He was successful in raising the contact angle of electrospun PVDF membranes from 80 °C to 154 °C by including hydrophobic clay nanoparticles into the polymer mat. From 98.2 to 99.9%, hydrophobic membranes demonstrated good salt rejection. Khayet et al. [182] investigated two-layer PVDF-polysulfone nanofiber membranes with various hydrophobic characteristics. It was discovered that when compared to single-layer membranes, two-layer membranes exhibit better water flux during desalination. Salt rejection was 99%, and the water fluxes of two-layer nanofiber membranes were 50,000 and 48,000 g/m^2^h at salt concentrations of 12 and 30 g/L. It has been determined that electrospinning is a trustworthy method for producing hydrophobic membranes [183]. This method of fabrication is superior to others due to its relatively low cost, flexibility in the use of different polymers and materials, and capacity to generate fibers with diameters ranging from several nanometers to several microns [116].

### 8.3 Membrane modification methods

The primary characteristic of a membrane that contributes significantly to improving the water flux is hydrophobicity. Although other membranes have less hydrophobic surfaces, practically yes, high contact angles (greater than 90°) are used. several techniques of modification, like the insertion of pore-forming substances [184], Inorganic nanoparticles and perfluorinated polymers were employed to enhance the hydrophobic characteristics and boost the water flux [185]. Simone et al. [186] modified microporous hydrophobic fibrous PVDF membrane. As a pore-forming agent, poly (vinylpyrrolidone) was used. Vacuum membrane distillation was used to test hydrophobic membranes with distilled water as the feed. At 50 °C and a vacuum pressure of 20 mbar, the water flux ranged from 3.5 to 18 kg/m^2^h.

The lack of water flux in salt purification and the need to maintain a vacuum are two drawbacks of this approach. A technique for creating hydrophilic-hydrophobic bilayer PVDF hollow fiber membranes utilizing silica as a hydrophobic modifier was developed by Edwie et al. [187]. The samples were examined in a sodium chloride and methanol DCMD solution. The salt rejection was more than 90%, and the highest water flux was 84 kg/m2/h. The hydrophilic-hydrophobic membrane had two layers, however it was unstable. Membrane surface alterations, which enable significant changes in membrane properties including roughness, hydrophobicity, and surface energy, are generally the most efficient modification techniques. Additionally, this method can be used to hydrophobize hydrophilic polymers. The simplest technique to immediately increase the hydrophobicity of membranes is to coat the membrane surface with a thin functional layer. A popular surface covering is sol-gel [188], and spinning, immersion [189].

One of the most effective ways to increase membrane hydrophobicity is graft polymerization. The membrane surface can change as a result of covalent bonds forming between the membrane and the grafted chains. Graft polymerization, as opposed to coating, increases the graft layer's chemical stability. It can therefore entirely eliminate the problem of hydrophobic layer instability. There are several ways to perform surface grafting, including photo- and thermally started graft polymerization of a single monomer or a mixture of two or more monomers, plasma- and radiation-induced graft polymerization, and more [190].

Plasma treatment is the process of ionized gas adsorption and polymerization onto the membrane surface. High-energy ions, reactive species, and photons produced during the discharge alter the polymer's surface. This method's primary benefit is the homogenous surface treatment it creates, which has a depth of several nanometers. When membrane distillation using hydrophobic membranes produced by plasma treatment was examined, the water flux ranged from 3 to 8 kg/m2/h at various monomer ratios, and salt rejection was greater than 98%. The qualities of the material can be greatly enhanced and varied by membrane modification. Convenience, ease of hardware design, and cost-effectiveness all play a role in the modification technique selected [178].

With electrospinning, you can have a lot of control over the membrane's porosity and thickness by adjusting the dope solution and spinning exposition time. Compared to other procedures like coating, its design calls for more durable equipment. On the other hand, employing only the dope solution on the membrane surface, it requires less material to change. The drop or spinning formation is determined by the viscosity of the dope solution, which allows the membranes to have a variety of various porosities and roughnesses [191]. The membrane needs to undergo a pretreatment to activate its structure in order to produce chemical interactions between the membrane and dope solution. The electrospun membranes exhibit high porosity, high hydrophobicity, low thickness, and high roughness. Because of its simple methodology and lack of complicated equipment, coating is the modification technique that has received the most research attention [192]. For instance, other than the standard lab equipment, the dip-coating procedure requires no additional equipment. A wide range of alterations are possible by changing the fluid in which the membrane is submerged. Through various crosslinks produced by grafting, coated membranes had varying degrees of hydrophobicity. The membrane porosity may be raised by dip coating with inorganic nanoparticle-containing solutions. Also, heavily fluorinated solutions are used to produce high LEPs. Vacuum filtration may produce highly homogenous surfaces, and the sol-gel technique can significantly alter the structure and mechanical strength of materials. However, the coating technique may adversely impact the membrane porosity and thickness because of the degree to which certain properties were under lower control during the experiment. Despite this, the coating technique appears to be the most practical for industrial applications because it is the simplest to fabricate among the related techniques and does not require high temperatures, pressures, or energies to design the process [191].

Membrane modifications open new doors for the recovery of water from industrial effluents by overcoming the MD process' operational constraints. For instance, utilizing modified membranes with hydrophilic chemicals makes it possible to recover water from oily solutions that would severely foul conventional membranes. Fluorinated polymers are modified in a way that allows for long-term operation and also improves wetting resistance. Inorganic nanoparticles, siloxanes and perfluoro polymers, and GO, respectively, can be used to operate with complex effluents in the presence of surfactant solutions, VOCs, and a number of inorganic foulants [193].

The main issue of membrane modification from a chemical engineering standpoint is to combine the desired feature demanded with materials economically viable through simpler and more efficient procedures. MD might become more prevalent in industrial processes thanks to effective and affordable membranes [194]. The duration of the MD process produced by employing the membrane is a crucial factor that needs to be taken into account for all membrane modification approaches. Scientific investigation into this subject is only getting started, and it is moving toward testing new modifications for a number of industrially necessary separations. As a prognosis, it should be noted that membranes will be improved using less complex techniques, like coating, to reduce MD operational issues in operations requiring huge volumes, like desalination or water recovery from industrial discharges [195].

In these situations, antifouling, long-term operating resistance, and self-cleaning capabilities will be desired and may be attained through coating. The use of more robust processes, such as electrospinning and plasma, where required characteristics may be swiftly and precisely obtained, is more common for separations involving high-value-added items that require strong membranes. The reduction of environmental concerns about water supply and water safety is also closely related to the evolution of membrane modification [196,197]. Table 6 [178], [125], [198], [199], [200], [189], [201], [202], [203], [204], [205], [206], [207], [208], provides summary of the modification methods and properties of membranes used in MD. The modification methods are radiation induced, plasma treatment, electrospinning, NIPS, sol-gel, Perfluorinated polymers method, phase inversion and UV graft method. The percentage salt rejection in case of radiation induced method is 99.98% while in case of electrospinning, plasma treatment, phase inversion, and Perfluorinated polymers, it is 99.9%, in sol -gel method, it is 99.3%, NIPS 99% and UV graft 97%. Highest value of flux is for NIPS (87,400 g/m^2^. h), and it is least for phase inversion (2900 g/m^2^. h). Highest contact angle (154) is observed for electrospinning, and least contact angle (92) is noted for phase inversion method.

**Table 6:** Modifications and properties of membranes used for MD [178].

Membrane	Methods	Contact angle	Rejection %	Flux g/m^2^.h	Reference
PES	Radiation-induced	114	99.98	50,500	[125]
PES	Plasma treatment	120	99.9	42,000	[198]
PVDF	Perfluorinated polymers	135	99.9	83,400	[199]
PVDF	Electrospining	130	99.9	49,000	[200]
PVDF	Electrospining	154	99	5800	[189]
PVDF	Electrospining	139	99.9	10,700	[201]
PVDF	NIPS	148	99	87,400	[202]
PS	Electrospining	113	99.9	31,000	[203]
SBS	Electrospining	132	99.9	10,500	[204]
PES	Sol-gel	119	99.3	44,700	[205]
PVDF	Phase inversion	92	99.9	2900	[206]
PVC	Radiation-induced	114	99.98	50,500	[207]
PP	UV graft	138	97	3000–8000	[208]

## 9 Conclusion and future outlook

Membrane distillation process is gaining popularity worldwide as a new and appealing water reclamation method, particularly for desalination and water treatment. Over the past 50 years, MD has experienced significant growth and offers a possible replacement for existing membrane separation techniques. The status and prospects of the MD process in desalination are covered in this review. This is a summary for the MD process where fundamental MD features, module configuration, and membrane properties were explored. Additionally, studies were performed on desalination technologies and desalination membranes. MD is an innovative desalination technique that has the potential to sustainably lessen the global water energy stress. When integrated with conventional seawater desalination systems like MSF, ED, RO MED, and FO, MD has shown significant potential in the desalination of saltwater/hypersaline water. The MD process can be made more affordable and sustainable by using low-cost energy sources like solar energy, geothermal energy, and waste heat salvaged from ships, industries, and power stations. The production of top-notch MD membranes with characteristics including high hydrophobicity, high permeation flux, low fouling propensity, great mechanical strength, low thermal conductivity, and high LEP can considerably aid in the commercialization of this technology. Weak mechanical properties of MD membranes, low water flux, and wetness are among obstacles that prevent MD from being widely commercialized. In this review work, it was also discovered that electrospinning is a potential technique for the fabrication of highly hydrophobic and highly porous effective MD membranes after taking into account current improvements in membrane fabrication techniques. Finally, electrospun membranes merit more attention. To advance and address various issues related to the MD process, studies and investigations are needed in this promising study area. In this respect, it is envisaged that electrospun membranes for MD applications would be commercialized.

## Appendix

The appendix contains all the abbreviations and their full names.

### Abbreviations Name

AG: Air gap
AGMD: Air gap membrane distillation
BTESE: Bis(triethoxysilyl)ethane
CNT: Carbon nanotube
CP: Concentration polarization
CVD: Chemical vapor deposition
DCMD: Direct contact membrane distillation
ED: Electrodialysis
ENM: Electrospun nanofibrous membrane
FO: Forward osmosis
FAS: Fluoroalkylsilanes
GO: Graphene oxide
LEP: Liquid entry pressure
MED: Multiple-effect distillation
MEE: Multiple effect evaporation
MSF: Multi-stage flash
MOF: Metal organic framework
MWCNT: Multi wall carbon nanotube
MoS_**2**_: Molybdenum disulfide
PVDF: Polyvinylidene fluoride
PTFE: Polytetrafluoroethylene
PES: Polyethersulfone
PE: Polyethylene
PP: Polypropylene
PFTS: Perfluorooctyltriethoxysilane
PSQ: Polysilsesquioxanes
RO: Reverse osmosis
SGMD: Sweep gas membrane distillation
TP: Temperature polarization
VMD: Vacuum membrane distillation
